# Engineering *Klebsiella sp*. 601 multicopper oxidase enhances the catalytic efficiency towards phenolic substrates

**DOI:** 10.1186/1471-2091-12-30

**Published:** 2011-05-31

**Authors:** Yadong Li, Zijun Gong, Xin Li, Yang Li, Xing-Guo Wang

**Affiliations:** 1 Faculty of Life Sciences, Hubei Unversity, Wuhan 430062 China

## Abstract

**Background:**

Structural comparison between bacterial CueO and fungal laccases has suggested that a charged residue Glu (E106) in CueO replaces the corresponding residue Phe in fungal laccases at the gate of the tunnel connecting type II copper to the protein surface and an extra α-helix (L351-G378) near the type I copper site covers the substrate binding pocket and might compromise the electron transfer from substrate to type I copper. To test this hypothesis, several mutants were made in *Klebsiella sp*. 601 multicopper oxidase, which is highly homologous to *E. coli *CueO with a similarity of 90% and an identity of 78%.

**Results:**

The E106F mutant gave smaller *K*_*m *_(2.4-7fold) and *k*_*cat *_(1-4.4 fold) values for all three substrates DMP, ABTS and SGZ as compared with those for the wild-type enzyme. Its slightly larger *k*_*cat*_*/K*_*m *_values for three substrates mainly come from the decreased *K*_*m*_. Deleting α-helix (L351-G378) resulted in the formation of inactive inclusion body when the mutant ^Δ^α351-378 was expressed in *E. coli*. Another mutant α351-380M was then made *via *substitution of seven amino acid residues in the α-helix (L351-G378) region. The α351-380M mutant was active, and displayed a far-UV CD spectrum markedly different from that for wild-type enzyme. Kinetic studies showed the α351-380M mutant gave very low *K*_*m *_values for DMP, ABTS and SGZ, 4.5-, 1.9- and 7-fold less than those for the wild type. In addition, *k*_*cat*_*/K*_*m *_values were increased, 9.4-fold for DMP, similar for ABTS and 3-fold for SGZ.

**Conclusion:**

The Glu residue at position 106 appears not to be the only factor affecting the copper binding, and it may also play a role in maintaining enzyme conformation. The α-helix (L351-G378) may not only block access to the type I copper site but also play a role in substrate specificities of bacterial MCOs. The α351-380M mutant catalyzing oxidation of the phenolic substrate DMP effectively would be very useful in green chemistry.

## Background

Multicopper oxidases (MCOs) contain a multiple copper center, and catalyze the oxidation of a variety of substrates along with the four-electron reduction of dioxygen to water [1]. Laccases are a member of family [2], and are widely distributed in plants and fungi [2,3]. Owing to their involvement in the transformation of a wide variety of phenolic compounds, including the polymeric lignin and humic substrates, fungal laccases attract considerable attention. It has been reported that laccases show multiple functions, including lignin degradation, pigmentation, lignin biosynthesis and pathogenesis in fungi, and, in addition to their organic substrate, require only molecular oxygen for catalysis, which makes them suitable for biotechnological application [4-11].

**Figure 1:**
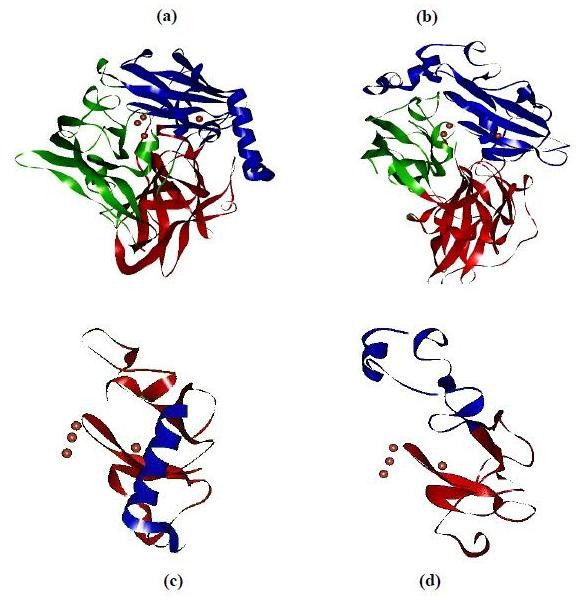
**Ribbon diagrams showing differences in substrate-binding sites between *Escherichia coli *(bacterium) and *Rigidoporus Lignosus *(fungus) multicopper oxidases**. Ribbon diagrams were created using PDB files deposited in the RCSB protein data bank *via *Swiss-PdbViewer 4.0.1. (a) *E. coli *CueO (PDB ID: 1KV7); (b) *R. Lignosus *laccase (PDB ID: 1V10); (c) The motif of *E. coli *CueO showing a α-helex (Leu351-Gly378) over the Cu I site; (d) The motif of *R. Lignosus *laccase showing the Cu I site exposed freely to solvent. Red ball presents copper ion in the active site of enzyme.

It has been reported recently that bacterial MCOs display laccase-like functions [12-15]. Several bacterial laccases were purified and crystallized, and two crystal structures, of *B. subtilis *CotA and *E. coli *CueO, were also determined at high resolution [16-18]. The structure of CueO, similar to that of CotA, is composed of three repeated pseudoazurin domains with the type I copper site in domain 3 and a trinuclear copper cluster between domains 1 and 3, very similar to the arrangement found in other laccases [16,17]. *E. coli *CueO is a 53.4 kDa periplasmic protein involved in the Cu efflux system and functions as the sole cupric oxidase responsible for the oxidation of cuprous ion to less toxic cupric ion whilst coupling four one-electron substrate oxidation steps to the reduction of dioxygen to water through a T1-type copper at the substrate-binding site and a trinuclear copper center at the oxygen-binding site [1,19-21]. However, the recombinant CueO is much less active than many fungal laccases. Several groups have performed studies to understand the relationship between structure and function of CueO, but as yet no clear conclusion has been reached up to date. Compared to fungal laccases, E106 perhaps decreases the micro-environmental hydrophobicity of the type II Cu, replacement of E106 by a phenylalanine (Phe) found at the corresponding site of fungal laccases slightly increases the oxidase activity of CueO [22]. Different from fungal laccases, an extra α-helix (L351-G378) near Cu I covers substrate binding pocket of CueO (see Figure 1). This α-helix might compromise the electron transfer from substrate to type I copper in bacterial MCOs [19].

In our previous study, the gene encoding a MCO in a soil bacterium *Klebsiella sp*. 601 was cloned, the enzyme, with a mass of 58.2 kDa was purified and a preliminary survey of the enzymatic properties of the recombinant MCO was carried out [15]. The amino acid sequence of *Klebsiella sp*. 601 MCO is highly homologous to that of *E. coli *CueO with a similarity of 90% and a 78% identity. High homology in amino acid sequences indicates two enzymes are likely to share essentially the same three-dimensional molecular structure. In this study, five mutants have been made *via *either amino acid deletion or replacement at E106 and the α-helix (L351-G378) region. Apart from three mutants expressing as inactive inclusion bodies, the mutants E106F and α351-380M with oxidase activities have been purified and studied enzymatically. Compatible with the result obtained in *E. coli *CueO, replacement of E106 by a Phe in *Klebsiella sp*. 601 MCO suggests that E106 is not the only factor affecting the copper binding. Alteration of amino acid residue in the mutant α351-380M changes the secondary structure of the α-helix, and significantly improves oxidase activity towards phenolic substrate DMP.

## Results

### Overexpression and purity of mutant enzymes

**Figure 2:**
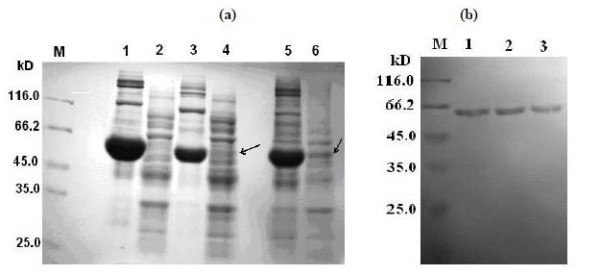
**15% SDS-PAGE showing overproduction and enzyme purity of three mutants of *Klebsiella sp. 601 *MCO**. (a) expressed products: M. protein molecular weight marker; lane 1. the pellet of E106F/α351-380M; lane 2. the supernatant of E106F/α351-380M; lane 3. the pellet of α351-380M; lane 4. the supernatant of α351-380M; lane 5. the pellet of E106F; lane 6. the supernatant of E106F. (b) purified proteins: lane 1. α351-380M, lane 2. E106F, and lane 3. the wild type.

Five mutated genes in the expression vector pET23a were separately transformed into *E. coli *BL21(DE3)pLysS to overproduce the respective mutant enzymes. In each case, *E. coli *transformants containing the pET23a recombinant plasmid with the mutated gene were cultured at 37°C in LB broth supplemented with 100 μg/ml ampicillin and then induced for 5 hours with 0.5 mM IPTG. A 15% SDS-PAGE showed abundant overproduction of all mutant enzymes, approximately equivalent to that seen with cloned wild-type enzyme under otherwise identical conditions. Unfortunately, the majority of overproduction was in pellets as inactive inclusion bodies, and only the wild-type and E106F enzymes showed about 10% and 5% soluble protein in supernatants. In order to obtain more soluble enzyme, temperatures for bacterial cultivation were optimized. When five transformants were cultured at 23°C, the mutants α351-380M, E106F and the wild type showed about 5-15% soluble enzyme, whilst the other mutants ^Δ^α351-378, E106F/^Δ^α351-378 and E106F/α351-380M still showed no visible band of over-produced protein in supernatants on 15% SDS gel. Table 2 summarizes overproduction of the five mutants and wild-type enzyme. All proteins in pellets and the crude extracts from three of the mutants, ^Δ^α351-378, E106F/Δα351-378 and E106F/α351-380M, gave negative reaction in the activity staining assay on 10% native gel (data not shown). Figure 2a shows a 15% SDS gel displaying overproduction of three mutants E106F, α351-380M and E106F/α351-380M under incubation at 23°C. The crude extract from the mutant E106F/α351-380M showed no obvious band at the expected position on the SDS gel, and also gave undetectable activity in the activity staining assay. Enzyme activities in crude extracts of five mutants and the wild-type were also measured spectrophotometrically, and the results are listed in Table 1.

**Table 1:** Protein expression of *Klebsiella sp. 601 *MCO and 5 mutants in *E. coli*

Mutation	Expression in *E.coli*	Active in supernatant	Protein in pellet
Wild type	+	+	+
E106F	+	+	++
^Δ^α351-378	+	-	++++
E106F/^Δ^α351-378	+	-	++++
α351-380M	+	+	+++
E106F/α351-380M	+	-	++++

Enzyme activity in supernatant was measured at 37°C by recording the change in A_447 _in 1 ml assay mixture containing 1 mM DMP, 0.2 mM CuSO_4_, 50 mM acetic acid-sodium acetate (pH5.0) and 10 μl crude extract. +: positive; -: negative.

**Table 2:** Comparison of kinetic parameters for three laccase substrates between two mutants and wild type of *Klebsiella sp. 601 *MCO

	Wild-type	E106F	α351-380M
DMP	pH5.0	pH5.0	pH5.0
K_m _(μM)	2,847 ± 30	1,176 ± 47	639 ± 17
k_cat _(s^-1^)	37,800 ± 32	37,480 ± 886	79,913 ± 944
k_cat_/K_m _(μM^-1^s^-1^)	13.27	31.87	125.06
ABTS	pH3.0	pH3.6	pH3.6
K_m _(μM)	539 ± 5.0	159 ± 1.0	284 ± 6.2
k_cat _(s^-1^)	68,409 ± 342	32,595 ± 152	35,990 ± 704
k_cat_/K_m _(μM^-1^s^-1^)	126.9	205.0	35,990 ± 704
SGZ	pH6.8	pH6.8	pH6.8
K_m _(μM)	11.67 ± 0.2	1.57 ± 0.02	1.46 ± 0.02
k_cat _(s^-1^)	10.8 ± 0.1	2.43 ± 0.02	4.3 ± 0.05
k_cat_/K_m _(μM^-1^s^-1^)	0.93	1.5	2.95

All data obtained from three independent experiments are presented as the mean of Michaelis-Menten parameters with the standard deviation (SD). Detailed measurements and data analysis were performed as described in Experimental section.

A Ni-affinity column was used for purifying the mutant and wild-type enzymes to minimize the risk of CueO contamination from *E. coli *host cells. After purification by Ni-affinity chromatography, the enzymes purified from the mutants E106F and α351-380M and the wild-type MCO exhibited a single band on the SDS gel with a molecular mass of about 60 kDa, similar to that (58.2 kDa) reported for wild-type enzyme [15] (see Figure 2b). About 15 mg E106F, 8 mg α351-380M and 20 mg wild-type pure enzymes were obtained respectively from 1L cultures of *E. coli *BL21(DE3)pLysS transformants after the purification described.

### Comparison of kinetic parameters between two mutant enzymes and wild-type MCO

Enzyme activities of two mutants and wild-type MCO were examined with a fixed concentration of DMP (1 mM), ABTS (0.5 mM) or SGZ (0.01 mM) over a range of pH values: the optimal pHs for the catalysis of two mutants were pH5.0 for DMP, pH3.6 for ABTS and pH6.8 for SGZ. The wild-type enzyme also gave the highest activities at pH 5.0 for DMP, pH3.0 for ABTS and pH6.8 for SGZ.

Kinetic parameters were estimated under the conditions described in the experimental section by varying the DMP (0.2-5.0 mM), ABTS (0.05-1.0 mM) and SGZ (0.6-20 μM) concentrations. The results are summarized in Table 2. As compared to the wild type, the *K*_*m *_value of E106F for DMP at pH5.0 is 2.4-fold low and the *k*_*cat *_value remains comparable. The decreased K_m _value results in a 2.4-fold increase in the catalytic coefficient (*k*_*cat*_*/K*_*m*_) in mutant E106F. The *K*_*m *_value for the α351-380M mutant is 4.5-fold smaller, and the *k*_*cat *_and *k*_*cat*_*/K*_*m *_values are 2.1-fold and 9.4-fold larger respectively, as compared with those for the wild-type enzyme. The large catalytic coefficient with a low *K*_*m *_value indicates the mutant α351-380M favors DMP as its substrate.

Kinetic parameters of two mutants for ABTS were also monitored at pH3.6. Both E106F and α351-380M mutants gave the *K*_*m *_values of 159 and 284 μM, 3.4- and 1.9-fold smaller than that (539 μM) for the wild-type enzyme at pH3.0. Nevertheless, the catalytic coefficients for both mutants are very close to that for the wild-type enzyme due to their low *k*_*cat *_values. Kinetic parameters for anther substrate SGZ were also estimated at pH6.8. Both mutants gave similar *K*_*m *_values for SGZ, 7-fold smaller than that for wild-type enzyme. However, again, *k*_*cat*_*/K*_*m *_values for both mutants are only 1.7- and 3-fold larger than that for the wild-type enzyme because of their low *k*_*cat *_values. Table 2 also shows that the wild-type enzyme, similar to the mutant E106F, catalyzes oxidation of ABTS much more effectively than the other two substrates DMP and SGZ. In contrast, the α351-380M mutant uses DMP as effectively as ABTS based on the values of *k*_*cat*_*/K*_*m *_obtained.

### Far-UV CD spectra of the two mutant enzymes

**Figure 3:**
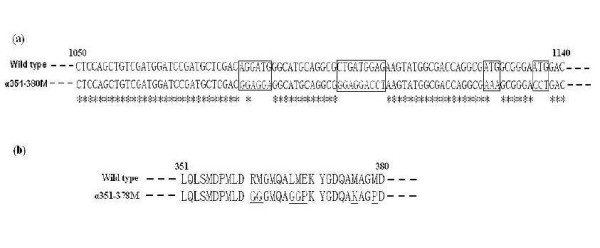
**Sequence comparison between the mutant α351-380M and the wild-type MCO**. (a) Nucleotide sequences; (b) Amino acid sequences. Those bases mutated in DNA sequence are framed and the corresponding amino acids changed in amino acid sequence are underlined.

**Figure 4:**
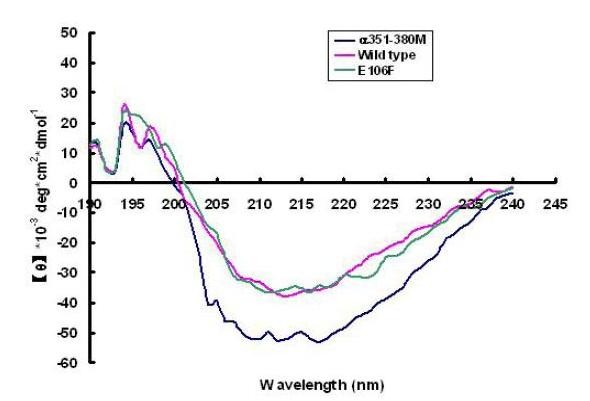
**Far-UV CD spectra of two mutants and the wild type of *Klebsiella sp. 601 *MCO**. Spectra were recorded at 20°C in 100 mM potassium phosphate, pH7.0.

In the mutant α351-380M, seven amino acid residues in the α-helix region were altered to abolish the secondary structure of the α-helix over the active pocket of the wild-type enzyme, based on the result of secondary structure prediction by the program PROF (http://www.aber.ac.uk/~phiwww/prof). PROF predicted that the mutation R361G/M362G./L367G/M368G/E369P/M376K/M379P could abolish α-helix structure at the α351-378 region. Figure 3 shows the detailed changes in both DNA and amino acid sequences of the mutant α351-380M. Although the program PROF predicted the α-helix structure the α351-378 region could unfold when the seven amino acid residues were substituted by those amino acids described, it needed to be tested whether this α-helix really vanished. To solve this issue, the far-UV CD spectra of two mutants and wild-type enzymes were recorded at 20°C in 0.1 M potassium phosphate (pH7.0), and the secondary structure content of each protein was estimated using the program JASCOW32. The far-UV CD spectrum at 208-228 nm for the α351-380M mutant was markedly less intense than that for the wild-type enzyme, while that of the point mutant E106F was essentially identical to the spectrum of the wild-type (Figure 4). The secondary structure prediction by the program JASCOW32 gave 16.9% α-helex, 23% β-sheet, 29.7% β-turn and 30.4% random coil for the wild-type enzyme, 17.1% α-helex, 22.8% β-sheet, 30% β-turn and 30.1% random coil for the mutant E106F, and 15.6% α-helex, 24.1% β-sheet, 30.1% β-turn and 30.2% random coil for the mutant α351-380M. CD spectrum analysis thus indicates that the E106F mutant may have similar secondary structure to the wild-type enzyme, whereas the replacement of seven amino acids in the α351-380M mutant appears to change α-helix structure as predicted by the computer program PROF.

## Discussion

Based on the information up to date, bacterial multicopper oxidases possess much lower oxidative activities compared to fungal laccases, although structure comparison between bacterial and fungal laccases shows the overall structure and copper coordination are very similar. Nevertheless, a charged residue Glu (E106) in CueO replaces the corresponding residue Phe in fungal laccases at the gate of the tunnel connecting type II copper to the protein surface [19]. Li et al (2007) have suggested the hydrophobic residue Phe in fungal laccases likely gates the tunnel and prevents type II copper exchange from solvent, while the charged residue Glu in CueO would make type II copper solvent accessible and easy to be lost [22]. According to this hypothesis, replacement of E106 by a Phe residue should enhance oxidase activities of *Klebsiella sp. 601 *MCO. Kinetic data collected from individual enzyme assay towards DMP, ABTS or SGZ shows that the E106F mutant has smaller *K*_*m *_and *k*_*cat *_values but larger *k*_*cat*_*/K*_*m *_values for all three substrates as compared with those for the wild-type enzyme (see Table 2). Its larger *k*_*cat*_*/K*_*m *_values for three substrates, which mainly comes from its small *K*_*m*_, indicate the mutant E106F catalyzes DMP, ABTS or SGZ more effectively than the wild-type enzyme. Although the *Km *value may not really represent binding constant for its substrate to an enzyme, smaller *K*_*m *_values for three substrates imply that E106 is not to be the only factor affecting the copper binding. Given that E106 is far from the substrate binding site, it is difficult to explain why E106F should have decreased *K*_*m *_values for all three substrates. Easy access of three substrates to the Cu I site in the active pocket may imply a possibly conformational change of this active pocket in the mutant enzyme. This may also offer us an explanation for the combined mutant E106F/α351-380M expressed in *E. coli *as inactive inclusion body owing to improper folding.

In the proposed mechanism of laccase catalysis, electrons are first transported from substrate to type I copper, and then travel through a 13 Å pathway formed by a cysteine and two histidines to type III copper, and finally reduce molecular oxygen to water at type II copper [23]. According to this mechanism, substrates should bind near the type I copper. In the molecular model of the complex of *B. subtilis *CotA and substrate, the ABTS-binding pocket is indeed very close to the type I copper site [16]. Kataoka et al (2007) also noticed that the substrate-binding site of CueO is buried deeply under a methionine-rich helical region including α-helices 5, 6 and 7, which is different from other MCOs [24]. They deleted the methionine-rich region Pro357-His406 and then replaced it with a Gly-Gly linker. The cuprous oxidase activity of the truncated mutant decreases to10% that of the wild-type enzyme, but the activities for laccase substrates ABTS and DMP are very similar in the presence of Cu(II) ions [24]. 50 amino acid residues replaced by a Gly-Gly linker may perhaps result in too much flexibility of the peptide chain containing rich Gly residues in solvent so that substrates may not easily gain access to the active pocket of the mutated enzyme. Detailed structural comparison has shown that the substrate binding pockets in fungal laccases are fully open and type I copper is exposed to solvent. In contrast, in the bacterial CueO, an extra α-helix (L351-G378) is located over type I copper and makes the substrate binding pocket smaller [19]. To test this hypothesis in the present study, a mutant ^Δ^α351-378 was first created by deleting 28 amino acids of the α-helix. The mutated protein was expressed in the form of inactive inclusion bodies in the pellet, similar to the result obtained in *E. coli *CueO [19]. The deletion of α-helix may result in improper folding of the ^Δ^α351-378 mutant. To further examine if the α-helix from L351 to G378 affects substrate binding, the mutant α351-380M was made through substitution of seven amino acid residues in the α-helix. Far-UV CD spectrum suggests that the replacement of seven amino acids appears to abolish the secondary structure of the α-helix between L351 G378. The α351-380M mutant is active towards all three substrates tested with very small *K*_*m *_values, 2-7 fold lower than those for the wild-type enzyme (see Table 2). Decreased *K*_*m *_values indicate that the α-helix from L351 to G378 indeed is a barrier for substrate binding to the enzyme. As compared to the wild-type enzyme, the α351-380M mutant gives a 2-fold lower *k*_*cat *_with a similar *k*_*cat*_*/K*_*m *_values for ABTS, a 2-fold lower *k*_*cat *_with a 3-fold larger *k*_*cat*_*/K*_*m *_value for SGZ, and a 2-fold larger *k*_*cat *_with a 10-fold larger *k*_*cat*_*/K*_*m *_value for DMP. Smaller *K*_*m*_, larger *k*_*cat *_and *k*_*cat*_*/K*_*m *_for DMP demonstrate that structural modification at α351-378 markedly enhances oxidase activity of *Klebsiella sp. 601 *MCO towards phenolic substrate DMP. The wild-type MCO favors ABTS as substrate, whereas the α351-380M mutant catalyzes the substrate DMP as effectively as ABTS, demonstrating that the α351-378 shifts its substrate specificity towards DMP. Our results suggest the α-helex may also play a role in substrate specificities of bacterial MCOs.

Laccases are very attractive enzymes for application in biotechnology and green chemistry. At present, only fungal laccases are used in industrial processes. However, fungal laccases are much more difficult to express as active enzymes in bacterial or yeast hosts to meet industrial requirement. Bacterial laccases perhaps are another candidate for industry. 10-fold increase of substrate specificity offers an opportunity to use the α351-380M mutant for biotechnological application. Its low soluble expression in *E. coli *will be improved in the near future by periplasmic protein expression, random mutagenesis or incubating under low temperature plus microaerobic conditions, because these strategies have been recently demonstrated to be a very useful in *B. licheniformis *and *Bacillus sp*. HR03 CotA [25,26]. The α351-380M mutant is an example of the successful modification of MCO function to a large extent, and extensive engineering of bacterial laccases is expected to create more novel functions.

## Conclusion

In summary, the data obtained from kinetic analysis of two mutants shows that (1) the glutamate residue at position 106 appears not to be the only factor affecting the copper binding, and it may also play a role in maintaining enzyme conformation correctly; and (2) the α-helix (L351-G378) may not only block access to the type I copper site but also play a role in substrate specificities of bacterial MCOs. The α351-380M mutant catalyzing oxidation of the phenolic substrate DMP effectively would be very useful in green chemistry.

## Materials and methods

### Materials and chemicals

KOD plus taq DNA polymerase, dNTP and Dpn1 were obtained from Takara and Toyoba (Japan). Laccase substrates 2,6-dimethylphenol (MDP), 2,2'-azino-bis-(3-ethylbenzibiozoline 6-sulfonic acid) (ABTS) and 4-hydroxy-3,5- dimethoxybenzaldehyde azine (SGZ) were acquired from Sigma (USA). Oligonucleotides for mutagenesis and DNA sequencing were made by Invitrogen (Shanghai). All chemicals were of analytical grade.

### Bacterial strains and vectors

The *E. coli *strains used in mutagenesis and gene expression were DH5α (F^- ^endA1 glnV44 thi-1 recA1 relA1 gyrA96 deoR nupG Φ80d*lacZ*ΔM15 Δ(*lacZYA-argF*)U169, hsdR17(r_K_^- ^m_K_^+^), λ^-^) and BL21(DE3)pLysS (F^- ^ompT gal dcm lon hsdS_B_(r_B_^- ^m_B_^-^) λ(DE3) pLysS(cm^R^)), both from Promega Biotech Co. The recombinant plasmid pET23a-601 harbouring *Klebsiella sp. 601 *MCO gene was constructed by Ms. Jiao Yin.

### Mutagenesis of Klebsiella sp. 601 MCO gene

Standard procedures [27] were used for extracting the plasmid pET23a-601 from the host strain *E. coli *DH5α, and transforming the plasmid DNA into *E. coli *DH5α for DNA amplification or *E. coli *BL21(DE3)pLysS for overexpression of the mutant enzymes. The strategy for mutagenesis, developed by Li *et al *(2008) [28], was modified and used for both base substitution and DNA deletion. The plasmid pET23a-601 DNA was used as a template, and the primers for mutagenesis (see table 3) were used for DNA amplification in the presence of KOD plus Taq DNA polymerase, MgCl_2 _and dNTP. The PCR reaction was initiated by preheating the reaction mixture at 94°C for 4 min, followed by 30 cycles of denaturation at 94°C for 15 s, annealing and extension at 68°C for 5 min with a final extension at 68°C for 8 min. To create the mutant α351-380M, two rounds of PCR were performed. In the first round, the plasmid pET23a-601 DNA, the oligonucleotides α351-380M-F1 and α351-380M-R1 were used as the template and primers for DNA amplification. In the second round, the plasmid DNA recovered from the first round of PCR was used as a template, and the oligonucleotides α351-380M-F2 and α351-380M-R2 were used as primers. imilar strategies were used to create E106F/^Δ^α351-378 and E106F/α351-380M *via *two or three rounds of PCR. The PCR-amplified DNA was separated by 0.7% agarose gel, recovered using the gel recovery kit (V-gene) and then digested with *Dpn*I at 37°C for 2 hours. The *Dpn*I-digested DNA was transformed into *E. coli *DH5α *via *the calcium chloride method [27], and positive colonies were selected at 37°C on the overnight-cultivated LB plates containing 100 μg/ml ampicillin. All mutants were screened and confirmed directly by DNA sequencing of the mutant genes.

**Table 3:** Oligonucleotide primers used for mutating *Klebsiella sp. 601 *MCO gene

Oligo name	Sequence
106F-F	5'-ACTGGCATGGCCTG**TTT**GTCCCGGGCGAGGTC-3'
106F-R	5'-CCTCGCCCGGGAC**AAA**CAGGCCATGCCAGTG -3'
^Δ^α351-378- F	5'- GGGCTGACGCAGCGTCAGATGGACCACGGCATGATGGA -3'
^Δ^α351-378- R	5'-CTGACGCTGCGTCAGCCCCGTCAGGGACGGT -3'
α351-380M-F1	5'-GATCCGATGCTCGAC**GGAGGA**GGCATGCAGGCG**GGAGGACCT**AAGTATGGC GACCAG-3'
α351-380M-R1	5'-CTGGTCGCCATACTT**AGGTCCTCC**CGCCTGCATGCC**TCCTCC**GTCGAGCAT CGGATC-3
α351-380M-F2	5'-TATGGCGACCAGGCG**AAA**GCGGGA**CCT**GACCACGGCATGATG -3'
α351-380M-R2	5'-CATCATGCCGTGGTC**AGG**TCCCGC**TTT**CGCCTGGTCGCCATA -3'

* Underlining indicates the sequences pairing between the forward and reverse primers for the deletion of α351-378. Mismatch bases are bold in each oligonucleotide.

In this study, five mutants were finally obtained through one, two or three rounds of PCR and *Dpn*I digestion as described above. In the mutant E106F, the phenylalanine codon (TTT) replaced the wild-type glutamate (GAG). To investigate if the α-helix (L351-G378) affects substrate access to the type I copper site, the mutant Δα351-378 was first created by deleting all 28 amino acids in the α351-378 region of wild-type enzyme. Then the mutant α351-380M was also made *via *substitution of 361R, 362M, 367L, 368M, 369E, 376M, 379M with a glycine, glycine, glycine, glycine, proline, lysine and proline, respectively. The point mutant E106F was also combined with the mutant ^Δ^α351-378 and α351-380M to form two other mutants E106F/^Δ^α351-378 and E106F/α351-380M. All mutations were confirmed by sequencing the overall DNA of each mutated gene.

### Enzyme preparation

The recombinant plasmids with the mutated MCO genes were extracted from *E. coli *DH5α, and then transformed into *E. coli *BL21(DE3)pLysS. Positive transformants of *E. coli *BL21(DE3)pLysS were grown at 23°C in LB broth containing 100 μg/ml ampicillin and induced by adding 0.5 mM isopropyl β-D-thiogalactopyranoside (IPTG) into the culture when OD_600 _was equal to 0.6. Bacterial cells were harvested and sonicated, and crude extracts were prepared through centrifugation at 27,000 g for 15 min.

Each mutant enzyme was purified by Ni-affinity chromatography, as described for the cloned wild-type MCO [15]. The clarified crude extract was loaded onto the Ni-column equilibrated with 100 mM phosphate (pH7.4). The column was washed with 100 mM phosphate (pH7.4) and then eluted with a gradient of 20-150 mM imidazole in 100 mM phosphate (pH7.4). Fractions were collected automatically with 1 ml per tube. Enzyme purity was routinely examined by SDS-PAGE on 15% gels, and the concentration of purified enzyme was estimated from A_280 _on the basis of the absorption coefficient of 1.03 mg ml^-1 ^predicted by the program ProtParam (http://expasy.org/tools/protparam.html). The pure enzyme, precipitated in 65% ammonium sulfate and stored at 4°C, was dialyzed before use against several changes of 100 mM phosphate buffer (pH7.4), and clarified by centrifugation.

### Enzyme assays

Enzyme activity in crude extract was initially examined using activity staining on 10% native gel, as described by Li et al [15]. The gel was first soaked in 50 mM acetate buffer (pH5.0) for 10 min, and then stained in 2.5 mM DMP, 0.2 mM CuSO_4 _and 50 mM H_3_PO_4_-Na_2_HPO_4 _(pH5.0) at room temperature for 10-15 min. Enzyme activity was also examined spectrophotometrically at 37°C by recording the change in A_447 _in the assay mixture containing 1 mM DMP, 0.2 mM CuSO_4_, 50 mM acetic acid-sodium acetate (pH5.0) and 10 μl crude extract.

Specific activity was measured with a Shimadzu UV/visible spectrophotometer (UV-2550). The extinction coefficients used in active assays were 14.8 mM^-1 ^cm^-1 ^for DMP, 36 mM^-1 ^cm^-1 ^for ABTS and 65 mM^-1 ^cm^-1 ^for SGZ. Oxidation of DMP was assayed at 37°C in 50 mM acetate (pH5.0), of ABTS in 50 mM acetic acid-sodium acetate (pH3.0 or 3.6), and of SGZ in 50 mM phosphate (pH6.8). In addition, 0.2 mM CuSO_4 _was routinely added into the reaction solution. Specific activities were expressed as units of activity per milligram of protein, where one unit represented a micromole of substrate oxidized per minute. To determine kinetic parameters, rates were measured spectrophotometrically at 37°C with 0.2 mM CuSO_4 _over a range of substrate concentrations (for DMP 0.2-5.0 mM, ABTS 0.05-1.0 mM and SGZ 0.6-20 μM). Michaelis-Menten parameters were calculated using the UVProbe-[Kinetics] version 1.11a (SHIMADZU Corporation), and kinetic parameters were determined by Lineweaver-Burk plot [29] and also checked by Hanes-Woolf and Eddie-Hofstee plots. The deviation between the same parameters obtained from different plots was less than 5%. In each plot, correlation coefficient (r^2^) value was equal to or large than 0.997. All data were also analyzed using the statistic software SPSS based on the non-linear regression method INVERSE, and analysis of variance gave P values of less than 0.005 in each case.

## Authors' contributions

All authors read and approved the final manuscript. X-GW conceived the study; YL, ZG and XL contributed to the design of the study; YL, ZG and XL performed the experiments and analyzed the results; X-GW and ZG wrote the paper.
